# What are the influencing factors on the outcome in lateral incisional hernia repair? A registry-based multivariable analysis

**DOI:** 10.1007/s10029-022-02690-y

**Published:** 2022-11-04

**Authors:** S. Schaaf, A. Willms, D. Adolf, R. Schwab, H. Riediger, F. Köckerling

**Affiliations:** 1 Department of General, Visceral and Thoracic Surgery, German Armed Forces Central Hospital Koblenz, Rübenacher Str. 170, 56072 Koblenz, Germany; 2 Department of General, Visceral and Vascular Surgery, Armed Forces Hospital Hamburg, Lesserstraße 180, 22049 Hamburg, Germany; 3 StatConsult GmbH, Am Fuchsberg 11, 39112 Magdeburg, Germany; 4 Hernia Center, Vivantes Humboldt Hospital, Academic Teaching Hospital of Charité, University Medicine, Am Nordgraben 2, 13509 Berlin, Germany

**Keywords:** Hernia surgery, Lateral hernias, Flank hernias, Lumbar hernias, Incisional hernia, Hernia registry, Outcome

## Abstract

**Introduction:**

Incisional hernias following lateral abdominal wall incisions with an incidence of 1–4% are less common than following medial incisions at 14–19%. The proportion of lateral incisional hernias in the total collective of all incisional hernias is around 17%. Compared to midline defects, lateral incisional hernias are more difficult to repair because of the more complex anatomy and localization. A recent systematic review identified only 11 publications with a total of 345 patients reporting on lateral incisional hernia repair. Therefore, further studies are urgently needed.

**Methods:**

Multivariable analysis of the data available for 6,306 patients with primary elective lateral incisional hernia repair was performed to assess the confirmatory pre-defined potential influence factors and their association with the perioperative and one-year follow-up outcomes.

**Results:**

In primary elective lateral incisional hernia repair, open onlay, open IPOM and suture procedures were found to have an unfavorable effect on the recurrence rate. This was also true for larger defect sizes and higher BMI. A particularly unfavorable relationship was identified between larger defect sizes and perioperative complications. Laparoscopic-IPOM presented a higher risk of intraoperative, and open sublay of postoperative, complications. The chronic pain rates were especially unfavorably influenced by the postoperative complications, preoperative pain and female gender.

**Conclusion:**

Open-onlay, open IPOM and suture procedures, larger defect sizes, female gender, higher BMI, preoperative pain and postoperative complications are associated with unfavorable outcomes following primary elective lateral incisional hernia repair.

## Introduction

Lateral abdominal wall hernias are defined as defects that occur within the anatomic region bounded by the costal margin, iliac crest, linea semilumaris and paraspinal muscles [1]. The European Hernia Society classifies lateral incisional hernias as subcostal (L1), flank (L2), iliac (L3) and lumbar (L4) hernias [2]. They occur most commonly following open renal procedures, retroperitoneal vascular procedures and following iliac bone harvesting [3]. Overall, incisional hernias following lateral abdominal wall incisions with an incidence of 1–4% are less common than following medial incisions at 14–19% [4].

The proportion of lateral incisional hernias in the total collective of all incisional hernias is around 17% [5].

Compared with midline defects, lateral incisional hernias are more difficult to repair because of the more complex anatomy and localization [3]. With a lower caseload, surgeons also generally lack the experience to repair lateral incisional hernias [3]. To date, there has also been a lack of guidelines for treatment of incisional hernias. An expert consensus guided by a systematic review contains no specific recommendations for treatment of lateral incisional hernias [6].

A systematic review collated the findings of 11 publications with a total of 345 patients following lateral incisional hernia repair [7]. Of these patients, only 27 (7.8%) underwent laparoscopic surgery. In these 11 studies the perioperative complication rates were 0–42%, recurrence rates 0–13% and the chronic pain rates 3–75% [7].

The authors conclude that the quality of the studies for repair of lateral incisional hernias is not sufficiently high to infer recommendations for everyday practice. Therefore, further studies are urgently needed for treatment of lateral incisional hernias.

The following analysis of data from the Herniamed Registry aims to analyze the influencing factors on the outcome of surgical repair of lateral incisional hernias.

## Methods

Herniamed is an internet-based hernia registry in which hospitals and independent surgeons in Germany, Austria and Switzerland can voluntarily document their routine hernia operations [8, 9]. The number of participating hospitals/surgical practices on 1 July 2021 was 816. All patients entered into the registry have signed a special consent form agreeing to their data being documented in the registry and to follow-up information being obtained after 1, 5 and 10 years. As part of the information provided to patients when giving informed consent to participate in the Herniamed Registry, they are also told that the treating hospital/ surgeon would like to be kept informed about any problem occurring after surgery. If problems or complications occur after the operation, the patient can at any time contact the treating hospital or treating surgeon to arrange for clinical examination. Perioperative complications are recorded for up to 30 days after surgery.

After 1, 5 and 10 years patients and their general practitioner are sent a questionnaire by the treating hospital or treating surgeon, enquiring once again about any postoperative complications.

In the questionnaire patients and their general practitioner are also asked about any pain at rest, pain on exertion or chronic pain requiring treatment. Patients and general practitioners are furthermore asked about any suspicious protrusion or recurrence.

If the patient or general practitioner reports chronic pain or suspected recurrence, the patient is invited by the treating hospital/surgeon for follow-up examination.

In the current analysis, the prospective data of patients who underwent primary elective lateral incisional hernia repair with the laparoscopic intraperitoneal onlay mesh (IPOM) technique or open suture, sublay, onlay or open IPOM approach were evaluated to assess all confirmatory pre-defined potential influencing factors on the perioperative and one-year follow-up outcome.

All analyses were performed with the software SAS 9.4 (SAS Institute Inc., Cary, NC, USA) and intentionally calculated to a full significance level of 5%, i.e. they were not corrected in respect of multiple tests, and each *p *value ≤ 0.05 represents a significant result.

Individual outcome and influencing variables (risk factors, complications) are summarized as global variables. A general, intra-or postoperative complication or risk factor is deemed present if at least one single item applies.

Unadjusted analyses were performed to analyze the effect of an individual influencing variable on an outcome parameter, with the main focus on the association with the surgical procedure. For a categorical outcome variable the chi-square test was used. For continuous outcome variables the ANOVA (analysis of variance) was used to analyze the influence of the comparison groups in, this case, four categories.

The relation of confirmatory pre-defined patient and procedure-related characteristics to the outcome parameters (general, intraoperative and postoperative complications, complication-related reoperations, recurrences as well as pain at rest, pain on exertion and pain requiring treatment after one year) was assessed using a binary logistic regression model that permitted simultaneous evaluation of several influencing parameters. In addition, all pair-wise odds ratios are given with the corresponding 95% confidence interval. This quantifies how changes in an influencing variable affected the likelihood estimation of the outcome variables, with the other parameters seen as constant. For the continuous influencing variable age the 10-year odds ratio is given and for body mass index (BMI) a 5-point odds ratio.

In addition to the surgical procedure (open direct suture/laparoscopic IPOM/open onlay/open sublay/open IPOM], the following are other potential influencing parameters:

1. Age in years
2. BMI in kg/m^2^
3. Gender [male/female]
4. ASA score [I/II/III–IV]
5. Defect size [W1 (< 4 cm)/W2 (> = 4–10 cm)/W3 (> 10 cm)]
6. Preoperative pain [yes/no/unknown]
7. Drainage [yes/no]
8. Mesh fixation [yes/no]
9. Presence of risk factors [yes/no]

as well as postoperative complications for analysis of pain at follow-up.

Risk factors apply if at least one of the following risk factors is present:

1. COPD
2. Diabetes mellitus
3. Aortic aneurysm
4. Immunosuppression
5. Corticoids
6. Smoking
7. Coagulopathy
8. Platelet aggregation inhibitors (discontinued less than 7 days ago)
9. Coumarin derivatives (Quick/INR not in normal range).

## Results

### Unadjusted analysis

**Fig. 1:**
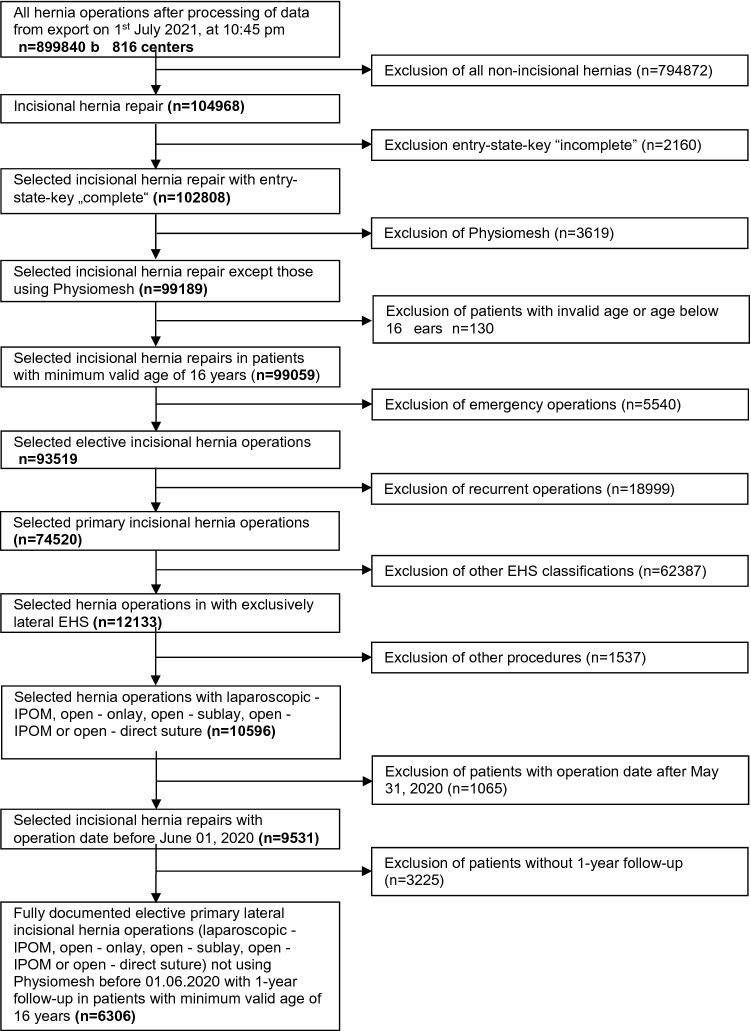
Flowchart of patient inclusion

As seen in the patient inclusion flowchart (Fig. 1), 6,306 patients with primary elective lateral incisional hernia repair were available for retrospective analysis of the prospectively collected data in the Herniamed Registry. All included patients were at least 16 years old and had 1 year follow-up. The surgical procedure performed was laparoscopic IPOM in 1,707 cases (27.07%), open sublay in 2,453 cases (38.90%), open suture in 877 cases (13.91%), open IPOM in 754 cases (11.96%) and open onlay in 515 cases (8.16%). Tables 1, 2 and 3 show the deviations in the frequency distribution of the potential influencing factors and outcome variables with respect to the surgical procedures. Table 1 shows the descriptive statistics as well as the test results for the continuous variables age and BMI and Table 2 the corresponding values for the categorical variables. Unadjusted analysis of the relationship between the surgical procedures and the patient- and diagnosis-related characteristics revealed that there were significant differences in all influencing variable scores. Table 3 shows unadjusted analysis of the relationship between the surgical procedures and the outcome variables.

**Table 1:** Presentation of ranges and unadjusted analysis results for homogeneity between operation procedures, age and BMI

		Surgical procedure					*p*
		Laparoscopic-IPOM	Open-onlay	Open-sublay	Open-IPOM	Open-direct suture	
Age [years]	*N*/mean ± sd	1707/64.7 ± 12.5	515/64.8 ± 12.8	2453/65.3 ± 12.0	754/65.6 ± 12.2	877/61.9 ± 15.3	< 0.001
BMI [kg/m^2^]	*N*/mean ± SD	1698/29.2 ± 5.5	513/28.8 ± 5.5	2443/28.7 ± 5.2	749/29.2 ± 5.5	875/27.4 ± 5.1	< 0.001

**Table 2:** Presentation of descriptive statistics and of unadjusted analysis results for homogeneity between operation procedures and categorical influencing variables

	Surgical procedure										*p*
	Laparoscopic-IPOM		Open-onlay		Open-sublay		Open-IPOM		Open-direct suture		
	*n*	%	*n*	%	*n*	%	*n*	%	*n*	%	
Gender											
Male	774	45.3	239	46.4	1202	49.0	392	52.0	340	38.8	< 0.001
Female	933	54.7	276	53.6	1251	51.0	362	48.0	537	61.2	
ASA											
I	180	10.5	49	9.5	224	9.1	65	8.6	154	17.6	< 0.001
II	1022	59.9	295	57.3	1401	57.1	421	55.8	490	55.9	
III/IV	505	29.6	171	33.2	828	33.8	268	35.5	233	26.6	
Defect size (incisional)											
I (< 4 cm)	705	41.3	200	38.8	850	34.7	269	35.7	736	83.9	< 0.001
II (4–10 cm)	842	49.3	259	50.3	1314	53.6	365	48.4	123	14.0	
III (> 10 cm)	160	9.4	56	10.9	289	11.8	120	15.9	18	2.1	
Preoperative pain											
No	522	30.6	158	30.7	733	29.9	259	34.4	241	27.5	0.008
Yes	1062	62.2	304	59.0	1516	61.8	455	60.3	559	63.7	
Unknown	123	7.2	53	10.3	204	8.3	40	5.3	77	8.8	
Drainage											
Yes	336	19.7	416	80.8	1856	75.7	427	56.6	312	35.6	< 0.001
No	1371	80.3	99	19.2	597	24.3	327	43.4	565	64.4	
Mesh											
Yes, fixation	1621	95.0	463	89.9	1874	76.4	725	96.2	15	1.7	< .001
Yes, no fixation	49	2.9	47	9.1	536	21.9	13	1.7	3	0.3	
No	37	2.2	5	1.0	43	1.8	16	2.1	859	97.9	
Risk factors-total											
Yes	649	38.0	209	40.6	981	40.0	317	42.0	309	35.2	0.035
No	1058	62.0	306	59.4	1472	60.0	437	58.0	568	64.8	
COPD											
Yes	137	8.0	34	6.6	254	10.4	82	10.9	73	8.3	0.007
No	1570	92.0	481	93.4	2199	89.6	672	89.1	804	91.7	
Diabetes											
Yes	215	12.6	73	14.2	297	12.1	114	15.1	92	10.5	0.048
No	1492	87.4	442	85.8	2156	87.9	640	84.9	785	89.5	
Aortic aneurysm											
Yes	7	0.4	6	1.2	28	1.1	10	1.3	4	0.5	0.035
No	1700	99.6	509	98.8	2425	98.9	744	98.7	873	99.5	
Immunosuppression											
Yes	24	1.4	9	1.7	63	2.6	18	2.4	14	1.6	0.077
No	1683	98.6	506	98.3	2390	97.4	736	97.6	863	98.4	
Corticoids											
Yes	25	1.5	3	0.6	41	1.7	13	1.7	16	1.8	0.399
No	1682	98.5	512	99.4	2412	98.3	741	98.3	861	98.2	
Smoking											
Yes	178	10.4	53	10.3	248	10.1	75	9.9	90	10.3	0.996
No	1529	89.6	462	89.7	2205	89.9	679	90.1	787	89.7	
Coagulopathy											
Yes	23	1.3	18	3.5	54	2.2	17	2.3	12	1.4	0.015
No	1684	98.7	497	96.5	2399	97.8	737	97.7	865	98.6	
Antithrombotic medication											
Yes	182	10.7	71	13.8	324	13.2	93	12.3	88	10.0	0.025
No	1525	89.3	444	86.2	2129	86.8	661	87.7	789	90.0	
Anticoagulant medication											
Yes	47	2.8	23	4.5	88	3.6	24	3.2	25	2.9	0.284
No	1660	97.2	492	95.5	2365	96.4	730	96.8	852	97.1

**Table 3:** Presentation of descriptive statistics and of unadjusted analysis results for homogeneity between procedures and outcome variables

		Surgical procedure										
		Laparoscopic - IPOM		Open - Onlay		Open - Sublay		Open - IPOM		Open - direct suture		
		*n*	%	*n*	%	*n*	%	*n*	%	*n*	%	*p*
Intraoperative complications - total	yes	31	1.8	2	0.4	23	0.9	16	2.1	19	2.2	0.003
	no	1676	98.2	513	99.6	2430	99.1	738	97.9	858	97.8	
General complications - total	yes	32	1.9	9	1.7	57	2.3	22	2.9	11	1.3	0.142
	no	1675	98.1	506	98.3	2396	97.7	732	97.1	866	98.7	
Postoperative complications - total	yes	64	3.7	31	6.0	164	6.7	37	4.9	37	4.2	<.001
	no	1643	96.3	484	94.0	2289	93.3	717	95.1	840	95.8	
Complication-related reoperations	yes	24	1.4	9	1.7	60	2.4	12	1.6	14	1.6	0.132
	no	1683	98.6	506	98.3	2393	97.6	742	98.4	863	98.4	
Recurrence on 1-year follow-up	yes	76	4.5	45	8.7	120	4.9	53	7.0	71	8.1	<.001
	no	1631	95.5	470	91.3	2333	95.1	701	93.0	806	91.9	
Pain on exertion on 1-year follow-up	yes	361	21.1	117	22.7	526	21.4	159	21.1	150	17.1	0.056
	no	1346	78.9	398	77.3	1927	78.6	595	78.9	727	82.9	
Pain at rest on 1-year follow-up	yes	200	11.7	71	13.8	321	13.1	94	12.5	85	9.7	0.073
	no	1507	88.3	444	86.2	2132	86.9	660	87.5	792	90.3	
Pain requiring treatment on 1-year follow-up	yes	164	9.6	60	11.7	236	9.6	67	8.9	66	7.5	0.129
	no	1543	90.4	455	88.3	2217	90.4	687	91.1	811	92.5	

Depending on the surgical procedure, the rates of recurrence were 4.5–8.7%, pain at rest 9.7–13.8%, pain on exertion 17.1–22.7% and of chronic pain requiring treatment 7.5–11.7%. The perioperative complications rates were 7.7–9.9%.

### Multivariable analyses

#### Intraoperative complications

The results of the model used for analysis of the association between the patient- and procedure-related characteristics with the occurrence of intraoperative complications are illustrated in Table 4 (model fit: *p* < 0.001). The risk of onset of intraoperative complications was significantly associated with the surgical procedures (*p* < 0.001), use of a drain (*p* = 0.003) and the defect size (*p* = 0.036) (Table 4).

**Table 4:** Multivariable analysis results for intraoperative complications, including odds ratio estimates with corresponding 95% confidence intervals

Variable	*P* value	Categories	Odds ratio	LCL	UCL	*P* value (pair-wise)
Surgical procedure	< 0.001	Open-sublay vs Laparoscopic-IPOM	0.298	0.158	0.564	< 0.001
		Open-IPOM vs Open-sublay	2.675	1.352	5.292	0.005
		Open-onlay vs Laparoscopic-IPOM	0.127	0.030	0.550	0.006
		Open-IPOM vs Open-onlay	6.263	1.424	27.534	0.015
		Open-onlay vs Open-direct suture	0.120	0.017	0.867	0.036
		Open-sublay vs Open-direct suture	0.281	0.065	1.209	0.088
		Open-onlay vs Open-sublay	0.427	0.100	1.832	0.252
		Open-IPOM vs Laparoscopic-IPOM	0.798	0.417	1.527	0.495
		Open-IPOM vs Open-direct suture	0.752	0.171	3.313	0.706
		Open-direct suture vs Laparoscopic-IPOM	1.061	0.251	4.494	0.936
Drainage	0.003	Yes vs no	2.102	1.278	3.455	
Defect size (incisional)	0.036	II (4–10 cm) vs I (< 4 cm)	1.879	1.114	3.167	0.018
		III (> 10 cm) vs I (< 4 cm)	2.185	1.068	4.470	0.032
		III (> 10 cm) vs II (4–10 cm)	1.163	0.629	2.149	0.630
Gender	0.061	Female vs Male	1.517	0.981	2.348	
Risk factors-total	0.127	Yes vs no	1.410	0.907	2.191	
ASA	0.228	III/IV vs II	1.510	0.944	2.415	0.085
		III/IV vs I	1.373	0.578	3.260	0.472
		II vs I	0.909	0.409	2.022	0.816
Age [10-years-OR]	0.347		0.917	0.765	1.099	
BMI [5-points-OR]	0.622		1.049	0.868	1.267	
Mesh	0.717	Yes, fixation vs Yes, no fixation	0.732	0.329	1.627	0.444
		Yes, fixation vs No	0.787	0.198	3.132	0.734
		Yes, no fixation vs No	1.075	0.230	5.014	0.927
Preoperative pain	0.773	Yes vs No	0.942	0.595	1.492	0.799
		Unknown vs no	0.703	0.269	1.840	0.473
		Yes vs unknown	1.339	0.530	3.381	0.536

**Fig. 2:**
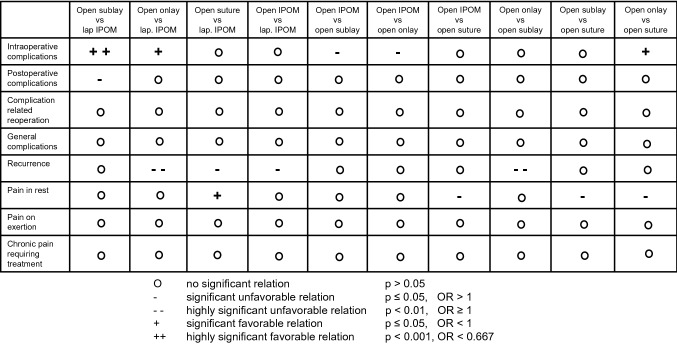
Relationship between outcomes and surgical procedures

Intraoperative complications were less common on comparing the open sublay and open onlay versus the laparoscopic and open IPOM procedures (Table 4, Fig. 2).

Intraoperative complications were less common in the open onlay than in the suture procedure (Table 4, Fig. 2).

**Fig. 3:**
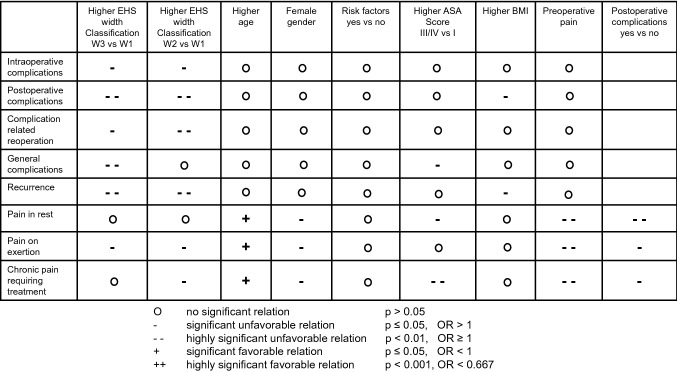
Relationship between outcomes and potential influencing factors

Larger defect sizes were also associated with a higher risk of intraoperative complications (Table 4, Fig. 3). Likewise, intraoperative complications were associated with a significantly more frequent use of drains (Table 4).

#### Postoperative complications

The results of the model used for analysis of the relation of the patient- and procedure-related characteristics to the risk of postoperative complications are shown in Table 5 (model fit: *p* < 0.001). Postoperative complications were significantly associated with the defect size (*p* < 0.001) and BMI (*p* = 0.002) (Table 5, Fig. 3). There was no global significant relation between the surgical procedure and the occurrence of postoperative complications (*p* = 0.153). Only in pair-wise comparisons the open sublay procedure compared with the laparoscopic IPOM was associated with a higher risk of postoperative complications (Table 5, Fig. 3).

**Table 5:** Multivariable analysis results for postoperative complications, including odds ratio estimates with corresponding 95% confidence intervals

Variable	*P* value	Categories	Odds ratio	LCL	UCL	*P* value (pair-wise)
Defect size (incisional)	< 0.001	III (> 10 cm) vs I (< 4 cm)	2.704	1.862	3.927	< 0.001
		II (4–10 cm) vs I (< 4 cm)	1.922	1.444	2.560	< 0.001
		III (> 10 cm) vs II (4–10 cm)	1.407	1.033	1.916	0.030
BMI [5-points-OR]	0.002		1.177	1.063	1.303	
Drainage	0.064	Yes vs No	1.308	0.985	1.736	
Risk factors-total	0.088	Yes vs No	1.228	0.970	1.555	
ASA	0.105	III/IV vs II	1.311	1.021	1.684	0.034
		III/IV vs I	1.206	0.745	1.953	0.445
		II vs I	0.920	0.588	1.440	0.715
Surgical procedure	0.153	Open-sublay vs Laparoscopic-IPOM	1.519	1.072	2.153	0.019
		Open-IPOM vs Open-Sublay	0.722	0.494	1.057	0.094
		Open-onlay vs Laparoscopic-IPOM	1.364	0.845	2.200	0.203
		Open-IPOM vs Open-onlay	0.805	0.488	1.327	0.394
		Open-sublay vs Open-direct suture	1.323	0.590	2.971	0.497
		Open-onlay vs Open-sublay	0.898	0.600	1.344	0.601
		Open-IPOM vs Laparoscopic-IPOM	1.097	0.710	1.697	0.676
		Open-onlay vs Open-direct suture	1.188	0.494	2.859	0.700
		Open-direct suture vs Laparoscopic-IPOM	1.148	0.499	2.642	0.746
		Open-IPOM vs Open-direct suture	0.956	0.405	2.256	0.918
Age [10-years-OR]	0.248		1.062	0.959	1.176	
Gender	0.282	female vs male	1.134	0.902	1.426	
Preoperative pain	0.645	Yes vs no	1.129	0.875	1.457	0.351
		Unknown vs no	1.108	0.709	1.731	0.654
		Yes vs unknown	1.019	0.671	1.548	0.929
Mesh	0.672	Yes, fixation vs No	0.713	0.338	1.502	0.373
		Yes, no fixation vs No	0.728	0.325	1.629	0.439
		Yes, fixation vs Yes, no fixation	0.980	0.682	1.408	0.912

#### Complication-related reoperations

The analysis results of the complication-related reoperations are presented in Table 6 (model fit: *p* < 0.001). The increased risk of a complication-related reoperation was significantly associated with the defect size (*p* = 0.002) and with the use of a drain (*p* = 0.032) (Table 6, Fig. 3).

**Table 6:** Multivariable analysis results for complication-related reoperations, including odds ratio estimates with corresponding 95% confidence intervals

Variable	*P* value	Categories	Odds ratio	LCL	UCL	*P* value (pair-wise)
Defect size (incisional)	0.002	II (4–10 cm) vs I (< 4 cm)	2.390	1.468	3.893	< 0.001
		III (> 10 cm) vs I (< 4 cm)	2.364	1.225	4.561	0.010
		III (> 10 cm) vs II (4–10 cm)	0.989	0.581	1.685	0.968
Drainage	0.032	Yes vs No	1.684	1.046	2.712	
ASA	0.090	III/IV vs II	1.512	1.000	2.286	0.050
		II vs I	0.639	0.329	1.240	0.186
		III/IV vs I	0.966	0.472	1.976	0.925
Mesh	0.144	Yes, fixation vs No	0.405	0.152	1.079	0.071
		Yes, no fixation vs no	0.529	0.180	1.559	0.248
		Yes, fixation vs Yes, no fixation	0.765	0.438	1.334	0.345
BMI [5-points-OR]	0.224		1.111	0.938	1.317	
Risk factors-total	0.507	Yes vs No	1.141	0.773	1.682	
Gender	0.669	Female vs male	0.922	0.634	1.339	
Preoperative pain	0.681	Yes vs no	0.964	0.640	1.453	0.861
		Yes vs unknown	0.755	0.403	1.415	0.381
		Unknown vs no	1.277	0.656	2.485	0.472
Surgical procedure	0.705	Open-sublay vs Open-direct suture	1.778	0.582	5.430	0.312
		Open-IPOM vs Open-sublay	0.732	0.384	1.398	0.345
		Open-onlay vs Open-sublay	0.737	0.360	1.507	0.402
		Open-direct suture vs Laparoscopic-IPOM	0.679	0.211	2.185	0.516
		Open-sublay vs Laparoscopic-IPOM	1.207	0.683	2.132	0.518
		Open-IPOM vs Open-direct suture	1.302	0.383	4.428	0.672
		Open-onlay vs Open-direct suture	1.310	0.363	4.719	0.680
		Open-IPOM vs Laparoscopic-IPOM	0.884	0.424	1.841	0.741
		Open-onlay vs Laparoscopic-IPOM	0.889	0.388	2.037	0.780
		Open-IPOM vs Open-onlay	0.994	0.412	2.402	0.990
Age [10-years-OR]	0.852		1.016	0.861	1.198	

#### General complications

The results of (the model used for) analysis of the influence exerted by the patient- and procedure-related characteristics on the risk of general complications are illustrated in Table 7 (model fit: *p* < 0.001). The general complications were significantly associated with the defect size (*p* < 0.001), ASA score (*p* = 0.002) and the use of drains (*p* = 0.012) (Table 7, Fig. 3).

**Table 7:** Multivariable analysis results for general complications, including odds ratio estimates with corresponding 95% confidence intervals

Variable	*P *value	Categories	Odds ratio	LCL	UCL	*P* value (pair-wise)
Defect size (incisional)	< 0.001	III (> 10 cm) vs II (4–10 cm)	2.326	1.489	3.633	< 0.001
		III (> 10 cm) vs I (< 4 cm)	2.482	1.486	4.144	< 0.001
		II (4–10 cm) vs I (< 4 cm)	1.067	0.693	1.644	0.768
ASA score	0.002	III/IV vs II	1.764	1.204	2.587	0.004
		III/IV vs I	5.199	1.557	17.367	0.007
		II vs I	2.947	0.906	9.587	0.073
Drainage	0.012	Yes vs no	1.778	1.136	2.782	
BMI [5-points-OR]	0.051		0.837	0.699	1.001	
Risk factors-total	0.217	Yes vs no	1.260	0.873	1.819	
Age [10-years-OR]	0.322		1.086	0.922	1.280	
Preoperative pain	0.493	Yes vs no	1.256	0.842	1.876	0.264
		Yes vs unknown	1.235	0.614	2.487	0.554
		Unknown vs no	1.017	0.482	2.144	0.965
Mesh	0.523	Yes, fixation vs no	2.475	0.497	12.323	0.269
		Yes, no fixation vs no	2.232	0.415	12.008	0.350
		Yes, fixation vs Yes, no fixation	1.109	0.603	2.040	0.740
Surgical procedure	0.594	Open-IPOM vs Open-onlay	1.740	0.785	3.857	0.173
		Open-onlay vs Laparoscopic-IPOM	0.607	0.274	1.346	0.219
		Open-onlay vs Open-direct suture	0.395	0.070	2.237	0.294
		Open-IPOM vs Open-sublay	1.290	0.765	2.173	0.340
		Open-onlay vs Open-sublay	0.741	0.362	1.519	0.414
		Open-sublay vs Open-direct suture	0.533	0.106	2.691	0.446
		Open-sublay vs Laparoscopic-IPOM	0.819	0.489	1.374	0.450
		Open-direct suture vs Laparoscopic-IPOM	1.536	0.298	7.924	0.608
		Open-IPOM vs Open-direct suture	0.688	0.131	3.606	0.658
		Open-IPOM vs Laparoscopic-IPOM	1.057	0.587	1.902	0.854
Gender	0.997	Female vs male	1.001	0.701	1.429	

#### Recurrence

The multivariable analysis results of the influencing factors on the onset of recurrences at one-year follow-up are given in Table 8 (model fit: *p* < 0.001). Larger defect sizes (*p* < 0.001) and higher BMI (*p* = 0.002) were associated with a higher recurrence risk (Table 8, Fig. 2). Laparoscopic-IPOM compared with open onlay, open IPOM and open suture procedure was found to present a significantly lower recurrence risk (Table 8, Fig. 2). The open sublay procedure posed a lower risk of recurrence than the open onlay procedure (Table 8, Fig. 2).

**Table 8:** Multivariable analysis results for recurrence, including odds ratio estimates with corresponding 95% confidence intervals

Variable	*P* value	Categories	Odds ratio	LCL	UCL	*P* value (pair-wise)
Defect size (incisional)	< 0.001	III (> 10 cm) vs I (< 4 cm)	2.097	1.457	3.020	< 0.001
		II (4–10 cm) vs I (< 4 cm)	1.541	1.186	2.002	0.001
		III (> 10 cm) vs II (4–10 cm)	1.361	0.986	1.879	0.061
Surgical procedure	< 0.001	Open-onlay vs Laparoscopic-IPOM	2.247	1.480	3.412	< 0.001
		Open-onlay vs Open-Sublay	1.960	1.363	2.819	< 0.001
		Open-IPOM vs Laparoscopic-IPOM	1.591	1.089	2.324	0.016
		Open-direct suture vs Laparoscopic-IPOM	2.410	1.069	5.432	0.034
		Open-sublay vs Open-direct suture	0.476	0.214	1.056	0.068
		Open-IPOM vs Open-sublay	1.388	0.975	1.975	0.069
		Open-IPOM vs Open-onlay	0.708	0.463	1.083	0.112
		Open-IPOM vs Open-direct suture	0.660	0.290	1.504	0.323
		Open-sublay vs Laparoscopic-IPOM	1.146	0.815	1.612	0.432
		Open-onlay vs Open-direct suture	0.932	0.403	2.155	0.870
BMI [5-points-OR]	0.002		1.172	1.062	1.292	
Gender	0.069	Female vs male	0.817	0.657	1.016	
Age [10-years-OR]	0.154		0.936	0.854	1.025	
Drainage	0.188	Yes vs no	0.842	0.652	1.088	
ASA score	0.512	III/IV vs I	1.296	0.833	2.017	0.251
		II vs I	1.193	0.803	1.773	0.383
		III/IV vs II	1.086	0.848	1.391	0.511
Mesh	0.694	Yes, fixation vs Yes, no fixation	0.852	0.585	1.240	0.402
		Yes, fixation vs No	0.916	0.428	1.964	0.822
		Yes, no fixation vs No	1.076	0.471	2.461	0.862
Risk factors-total	0.832	Yes vs no	1.025	0.816	1.288	
Preoperative pain	0.927	Yes vs no	1.015	0.799	1.288	0.906
		Yes vs unknown	1.085	0.717	1.643	0.698
		Unknown vs no	0.935	0.603	1.448	0.762

#### Pain at rest

The multivariable analysis results of the influencing factors on pain at rest at one-year follow-up are illustrated In Table 9 (model fit: *p* < 0.001).

**Table 9:** Multivariable analysis results for pain at rest, including odds ratio estimates with corresponding 95% confidence intervals

Variable	*P* value	Categories	Odds ratio	LCL	UCL	*P* value (pair-wise)
Age [10-years-OR]	< 0.001		0.812	0.761	0.866	
Preoperative pain	< 0.001	Yes vs no	1.641	1.362	1.978	< 0.001
		Unknown vs no	1.351	0.982	1.858	0.065
		Yes vs Unknown	1.215	0.909	1.624	0.188
Postoperative complications-total	< 0.001	Yes vs no	1.783	1.337	2.376	
Gender	0.007	Female vs male	1.243	1.062	1.455	
ASA score	0.007	III/IV vs I	1.628	1.190	2.227	0.002
		III/IV vs II	1.213	1.014	1.451	0.034
		II vs I	1.342	1.017	1.771	0.038
Surgical procedure	0.076	Open-onlay vs Open-direct suture	2.311	1.258	4.246	0.007
		Open-sublay vs Open-direct suture	2.124	1.210	3.729	0.009
		Open-IPOM vs Open-direct suture	2.057	1.142	3.704	0.016
		Open-direct suture vs Laparoscopic-IPOM	0.539	0.306	0.949	0.032
		Open-onlay vs Laparoscopic-IPOM	1.246	0.909	1.706	0.171
		Open-Sublay vs Laparoscopic-IPOM	1.145	0.917	1.430	0.233
		Open-IPOM vs Laparoscopic-IPOM	1.108	0.843	1.457	0.461
		Open-IPOM vs Open-onlay	0.890	0.634	1.250	0.500
		Open-onlay vs Open-sublay	1.088	0.820	1.443	0.558
		Open-IPOM vs Open-sublay	0.968	0.748	1.254	0.806
Defect size (incisional)	0.178	II (4–10 cm) vs I (< 4 cm)	1.177	0.987	1.405	0.070
		III (> 10 cm) vs I (< 4 cm)	1.177	0.890	1.555	0.254
		III (> 10 cm) vs II (4–10 cm)	0.999	0.771	1.295	0.996
Mesh	0.258	Yes, fixation vs No	0.651	0.390	1.086	0.100
		Yes, no fixation vs No	0.666	0.381	1.164	0.154
		Yes, fixation vs Yes, no fixation	0.977	0.757	1.262	0.861
Drainage	0.388	Yes vs no	0.922	0.768	1.108	
Risk factors-total	0.450	Yes vs no	0.938	0.795	1.107	
BMI [5-points-OR]	0.731		1.013	0.943	1.088	

A lower risk of pain at rest at one-year follow-up was found in higher age (*p* < 0.001) (Table 9, Fig. 3). Preoperative pain (*p* < 0.001), postoperative complications (*p* < 0.001), female gender (*p* = 0.007) and higher ASA score presented a higher risk of pain at rest (*p* = 0.007) (Table 9, Fig. 3). Surgical procedures tended to be associated with at rest (*p* = 0.076).

In detail, only the open suture procedure compared with laparoscopic and open IPOM, open onlay and open sublay presented a lower pain risk (Table 9, Fig. 2).

#### Pain on exertion

The multivariable analysis results of pain on exertion at one-year follow-up are shown in Table 10 (model fit: *p* < 0.001).

**Table 10:** Multivariable analysis results for pain on exertion, including odds ratio estimates with corresponding 95% confidence intervals

Variable	*P* value	Categories	Odds ratio	LCL	UCL	*P* value (pair-wise)
Age [10-years-OR]	< 0.001		0.770	0.730	0.811	
Preoperative pain	< 0.001	Yes vs no	1.562	1.346	1.813	< 0.001
		Unknown vs no	1.712	1.340	2.189	< 0.001
		Yes vs unknown	0.912	0.730	1.141	0.421
Gender	< 0.001	Female vs male	1.431	1.259	1.627	
Defect size (incisional)	0.004	II (4–10 cm) vs I (< 4 cm)	1.241	1.075	1.433	0.003
		III (> 10 cm) vs I (< 4 cm)	1.355	1.083	1.696	0.008
		III (> 10 cm) vs II (4–10 cm)	1.092	0.886	1.345	0.410
Postoperative complications-total	0.040	Yes vs No	1.313	1.012	1.702	
ASA score	0.384	III/IV vs II	1.101	0.949	1.276	0.203
		III/IV vs I	1.148	0.900	1.465	0.266
		II vs I	1.043	0.844	1.289	0.696
Drainage	0.423	Yes vs no	1.063	0.916	1.234	
BMI [5-points-OR]	0.479		1.021	0.963	1.083	
Mesh	0.518	Yes, fixation vs no	1.318	0.811	2.140	0.265
		Yes, no fixation vs no	1.272	0.756	2.138	0.365
		Yes, fixation vs Yes, no fixation	1.036	0.838	1.281	0.744
Risk factors-total	0.628	Yes vs no	1.034	0.904	1.183	
Surgical procedure	0.986	Open-onlay vs Open-sublay	1.069	0.846	1.350	0.579
		Open-onlay vs Laparoscopic-IPOM	1.043	0.806	1.349	0.751
		Open-onlay vs Open-direct suture	1.089	0.630	1.882	0.761
		Open-IPOM vs Open-onlay	0.961	0.727	1.270	0.778
		Open-sublay vs Laparoscopic-IPOM	0.976	0.815	1.168	0.789
		Open-IPOM vs Open-sublay	1.026	0.831	1.268	0.809
		Open-IPOM vs Open-direct suture	1.046	0.615	1.779	0.869
		Open-direct suture vs Laparoscopic-IPOM	0.958	0.572	1.604	0.870
		Open-sublay vs Open-direct suture	1.019	0.610	1.702	0.943
		Open-IPOM vs Laparoscopic-IPOM	1.002	0.803	1.250	0.989

Higher age was associated with a lower risk of pain on exertion (*p* < 0.001). Preoperative pain (*p* < 0.001), female gender (*p* < 0.001), larger defect size (*p* = 0.004) and postoperative complications (*p* = 0.040) were associated with a significantly higher rate of pain on exertion (Table 10, Fig. 3).

#### Chronic pain requiring treatment

The multivariable analysis results of the influencing factors on chronic pain requiring treatment following lateral incisional hernia repair are illustrated in Table 11 (model fit: *p* < 0.001). Here, too, a lower risk was identified in higher age (*p* < 0.001).

**Table 11:** Multivariable analysis results for pain requiring treatment, including odds ratio estimates with corresponding 95% confidence intervals

Variable	*P* value	Categories	Odds ratio	LCL	UCL	*P* value (pair-wise)
Age [10-years-OR]	< 0.001		0.800	0.744	0.859	
Preoperative pain	< 0.001	Yes vs no	1.764	1.420	2.191	< 0.001
		Unknown vs no	1.723	1.218	2.437	0.002
		Yes vs unknown	1.024	0.752	1.394	0.881
Gender	< 0.001	Female vs male	1.356	1.136	1.620	
Postoperative complications-total	0.002	Yes vs no	1.681	1.219	2.318	
ASA	0.003	III/IV vs I	1.900	1.308	2.760	< 0.001
		II vs I	1.668	1.193	2.332	0.003
		III/IV vs II	1.139	0.932	1.392	0.202
Defect size (incisional)	0.061	II (4–10 cm) vs I (< 4 cm)	1.266	1.038	1.544	0.020
		III (> 10 cm) vs II (4–10 cm)	0.873	0.648	1.175	0.370
		III (> 10 cm) vs I (< 4 cm)	1.105	0.802	1.523	0.543
BMI [5-points-OR]	0.333		1.040	0.961	1.125	
Risk factors-total	0.466	Yes vs no	1.071	0.891	1.287	
Operationsmethode	0.547	Open-onlay vs Open-sublay	1.263	0.928	1.718	0.138
		Open-IPOM vs Open-onlay	0.768	0.526	1.121	0.171
		Open-onlay vs Open-direct suture	1.555	0.754	3.207	0.232
		Open-onlay vs Laparoscopic-IPOM	1.195	0.849	1.682	0.306
		Open-direct suture vs Laparoscopic-IPOM	0.769	0.388	1.525	0.452
		Open-sublay vs Open-direct suture	1.231	0.622	2.436	0.550
		Open-IPOM vs Laparoscopic-IPOM	0.918	0.672	1.254	0.591
		Open-IPOM vs Open-direct suture	1.194	0.586	2.432	0.625
		Open-sublay vs Laparoscopic-IPOM	0.946	0.738	1.214	0.665
		Open-IPOM vs Open-sublay	0.970	0.719	1.308	0.840
Drainage	0.896	Yes vs No	1.014	0.825	1.247	
Mesh	0.946	Yes, fixation vs Yes, no fixation	0.953	0.715	1.270	0.742
		Yes, no fixation vs No	1.027	0.518	2.035	0.939
		Yes, fixation vs no	0.979	0.518	1.849	0.947

Likewise, preoperative pain (*p* < 0.001), postoperative complications (*p* = 0.002) and female gender (*p* < 0.001) were found to present a significantly higher risk of chronic pain requiring treatment (Table11, Fig. 3). This was also true for higher ASA score (*p* = 0.003).

## Discussion

The most important study so far on the treatment of lateral incisional hernia is the review by Zhou et al. [7] which included 11 publications with a total of 345 lateral incisional hernia repairs. That review reported on the perioperative complication rates and findings after 4–60 months’ follow-up. In the Herniamed analysis presented here all patients were followed up for one year.

Whereas the rate of laparoscopic operations in the review was only 7.8%, in the Herniamed Registry it was 27.1%. It is not possible to give a more precise description of the open surgical procedures used in the review, but the predominant procedure was the open retromuscular/sublay procedure [7]. In the Herniamed Registry, too, the open retromuscular/sublay procedure at 38.9% was the most commonly used surgical procedure.

The unadjusted analysis results for the different surgical procedures revealed for the Herniamed cohort recurrence rates of 4.5–8.7% and for pain on exertion 17.1–22.7%. As such, the unadjusted results for the various surgical procedures are in the same range reported in the review.

**Fig. 4:**
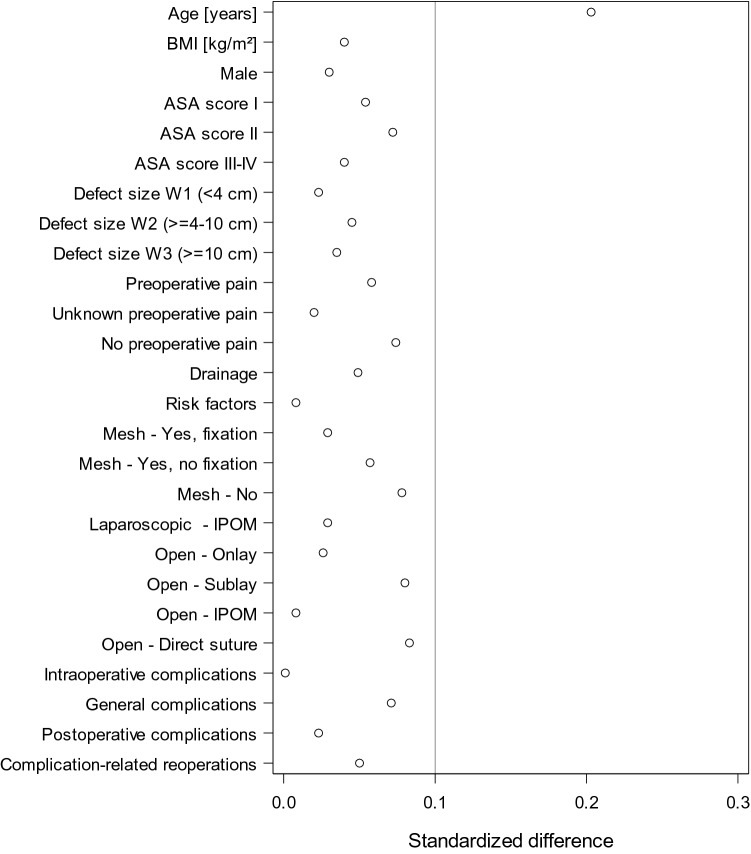
Standardized differences

In the review no further analysis was carried out to identify the influence exerted by the choice of surgical procedure and the presence of other potentially influencing factors on the outcome of lateral incisional hernia repair (Fig. 4).

The intraoperative complication rate was highly significantly or significantly unfavorably associated with the laparoscopic and open IPOM, open suture procedure and larger defect sizes.

The postoperative complication rate revealed a very unfavorable or unfavorable relation to the open retromuscular/sublay procedure, larger defect size and higher BMI. The complication-related reoperation rate was associated unfavorably or very unfavorably only with larger defect sizes.

The general complications were found to have a very unfavorable relationship with defect sizes > 10 cm and an unfavorable association with higher ASA score.

The recurrence rate was significantly or highly significantly unfavorably related to the open onlay surgical procedure, suture procedure and open IPOM as well as by larger defect sizes and higher BMI.

The rates of pain on exertion and chronic pain requiring treatment were not significantly related to the surgical procedures. Only for pain at rest was a significantly more favorable association identified for the use of the suture procedure. A highly significantly or significantly unfavorable relation was found between the pain rates and defect sizes, female gender, higher ASA score, preoperative pain and postoperative complications. Higher age had a significantly favorable association with the pain rates.

It can thus be concluded that for the choice of surgical procedure in lateral incisional hernia repair the risk of chronic pain tends to be of lesser importance. Chronic pain was found to be significantly more unfavorably related to larger defect sizes, younger age, female gender, higher ASA score, preoperative pain and postoperative complications. But for the recurrence rate a significantly or highly significantly increased risk was identified for the open suture procedure, open onlay procedure and open IPOM procedure. Hence, in terms of recurrence rate the more favorable outcomes were identified for the laparoscopic IPOM procedure and the open retromuscular/sublay procedure. Therefore, the defect size and BMI, which had a significant to highly significant relation to the recurrence rate, must also be taken into account. Furthermore, the laparoscopic IPOM presented a higher risk of intraoperative complications and the open retromuscular/sublay procedure of postoperative complications. Hence, it appears that the ideal surgical procedure for lateral incisional hernias has not been identified so far.

Intraperitoneal mesh placement has been the subject of increasing controversial debate in recent years because of potential complications [10]. Therefore, there has been a sharp decline in number of laparoscopic IPOM procedures for treatment of incisional hernias [11]. This has led to an increase in the use of the open retromuscular/sublay procedure, which is associated with a significantly higher rate of perioperative complication rates [11]. The future alternative to the laparoscopic IPOM and open retromuscular/sublay procedure for repair of lateral incisional hernias is likely to be laparo-endoscopic or robotic transversus abdominis release procedures or hybrid techniques [12–16]. Initial, promising results are available for the procedures. The results must be measured against those presented here for the established surgical procedures.

Of course, results based on registry data bear some risk of bias and are potentially hampered by missing or incorrect data [5]. Thus, all responsible surgeons participating in the Herniamed Registry sign a contract for data correctness and completeness [5]. The Registry software will flag up any missing data. At one-year follow-up postoperative complications are queried once again. On certification of hernia centers the experts can check the data for completion and correctness. The lack of follow-ups (drop-out) for a relevant proportion is another limitation of the registry, but the subgroup analysis does not show any selection bias.

In summary, it can be stated that the open onlay, open IPOM and suture procedures have an unfavorable relation to the recurrence rate. Hence, for repair of primary lateral incisional hernias preference should be given to the laparoscopic IPOM and the open sublay procedure. As regards the risk of chronic pain no significant difference has been identified between the procedures. The laparoscopic IPOM is associated with a higher risk of intraoperative, and the open sublay procedure of postoperative, complications. Larger defect sizes are found to have the most unfavorable association with the perioperative complication rates and recurrence rates. The most unfavorable relationship with chronic pain is seen for postoperative complications, female gender and preoperative pain. Higher age has a favorable association with chronic pain.
